# Can Artificial Intelligence Really Help? A Practicing Radiologist’s Simplified Guide to AI, with a Critical Appraisal of the Use of AI in Cancer-Associated Thromboembolism

**DOI:** 10.3390/cancers18172753

**Published:** 2026-08-25

**Authors:** Julia H. Miao, Ola A. E. Mohamed, Christopher Straus, Vanessa Peters, Emily Miller, Basant Dawoud, Joshua Brooks, Haidy Megahed, Ahmed Hamimi

**Affiliations:** 1 Department of Radiology, University of Chicago, Chicago, IL 60637, USA; 2 Department of Radiology, Mayo Clinic, Rochester, MN 55905, USA; 3 Department of Radiology, Tanta University, Tanta 31527, Egypt; 4 Department of Family and Community Medicine, Texas Tech University Health Sciences Center, Lubbock, TX 79430, USA

**Keywords:** artificial intelligence, cancer-associated thrombosis, evidence grading, pulmonary embolism, risk prediction

## Abstract

Artificial intelligence (AI) has generated considerable excitement in radiology, with claims of transformative improvements in diagnostic accuracy, workflow efficiency, and clinical decision support. However, a critical gap persists between AI’s theoretical promise and its real-world performance, particularly in complex, high-stakes scenarios such as cancer-associated thromboembolism (CAT). CAT is a leading cause of morbidity and mortality in oncology patients, yet it remains underdiagnosed on routine imaging. This paper provides a general radiology critique of current AI applications, then narrows focus to CAT management. This review additionally evaluates AI’s role in incidental pulmonary embolism detection, risk stratification, and treatment decision support. While AI demonstrates sensitivity gains, it faces substantial limitations: data heterogeneity, lack of prospective validation, poor generalizability across cancer subtypes, and integration challenges with clinical workflows. Therefore, AI is not yet a reliable standalone tool for CAT management, but may serve as an adjunct if clinically validated, explainable, and embedded within multidisciplinary frameworks.

## Simple Summary

This paper looks at whether artificial intelligence (AI) can help doctors detect and manage blood clots in cancer patients—a serious condition called cancer-associated thromboembolism (CAT). The authors explain that while AI shows promise for finding clots on CT scans, it often gives false alarms, especially in cancer patients. AI also cannot reliably tell tumor clots from ordinary clots, and it has not been proven to improve patient survival. The authors conclude that AI is not ready to work alone. Instead, it should serve as a helpful second pair of eyes for radiologists, with human oversight still absolutely necessary.

## 1. Introduction

AI is being introduced into all aspects of our lives and our practice, promising a monumental advancement in medical practice. Technological advancements in deep learning and computer performance have enabled machines to identify complex patterns in medical images with increasing accuracy, and in some cases, claim superhuman performance [1,2]. As of 1 July 2026, there were 1524 FDA-approved AI tools in medicine. Among them, there were 1164 tools for radiology, accounting for almost 76% [3]. By any measure, radiology has become the de facto home of medical AI. Yet a striking paradox exists. Walk the floor of the Radiological Society of North America (RSNA) annual meeting, and you will encounter nearly 3000 AI vendors, each promising transformative change in radiology practice. Step back into the reading room, however, and the reality is far more modest. Widespread clinical adoption remains limited, and a significant proportion of practicing radiologists report limited familiarity with AI development pipelines, validation methodology, and the practical criteria by which a tool should be selected and evaluated. In short, radiology may own AI—but radiologists, by and large, do not yet own their relationship with it. However, radiologists face a critical problem: separating hype from evidence. Vendor claims often outpace published data. Nowhere is this gap more consequential than in cancer-associated thromboembolism (CAT), where AI is heavily promoted for detecting pulmonary emboli, predicting thrombotic risk, and distinguishing bland from tumor thrombus.

This review has two goals. First, we provide a practical overview of FDA-approved AI tools across radiology for radiologists/oncologists with limited AI knowledge. Second, we offer an evidence-based critique of AI specifically for CAT, grading evidence from Level I (meta-analyses) to Level V (expert opinion) and answering the question: “Can AI help?”

## 2. History of AI in Radiology

The history of artificial intelligence in radiology reflects a steady evolution from early rule-based systems to today’s advanced deep learning algorithms capable of rivaling human performance in specific imaging tasks. The concept of Computer-Aided Diagnosis (CAD) started to emerge in the 1980s. Tasks like edge detection and thresholding started to appear, but it was markedly limited by computing power and lack of digital imaging infrastructure. The first FDA-approved tool was a CAD system for mammography in 1995. An explosion of digital imaging data and computer power through enhanced GPUs started in the 2010s, leading to the introduction of machine learning principles and Convolutional Neural Networks (CNNs), with the first studies applying the concept of deep learning starting to appear. In the period between 2016 and 2020, there was an increasing integration of AI tools into the PACS and radiology workflow, with robust application of clinical validation and regulatory approvals. From 2020 to now, there has been broader adoption in hospital and teleradiology platforms. Multimodal AI combining text, image, and labs is starting to kick in. Currently, there is an increasing focus on explainability, bias mitigation, and human-AI collaboration.

## 3. Defining AI in the Radiologic Context

Artificial intelligence (AI) differs fundamentally from traditional rule-based software: AI learns patterns from data rather than following static instructions, and it can be retrained to improve performance. Key subfields include machine learning (ML), where algorithms identify predictive features from examples (e.g., detecting lung nodules), and deep learning (DL), a subset of ML that uses multi-layer neural networks for tasks such as organ segmentation. DL underpins most advanced radiology AI applications today.

## 4. The Radiology Life Cycle as an AI Integration Landscape

**Figure 1:**
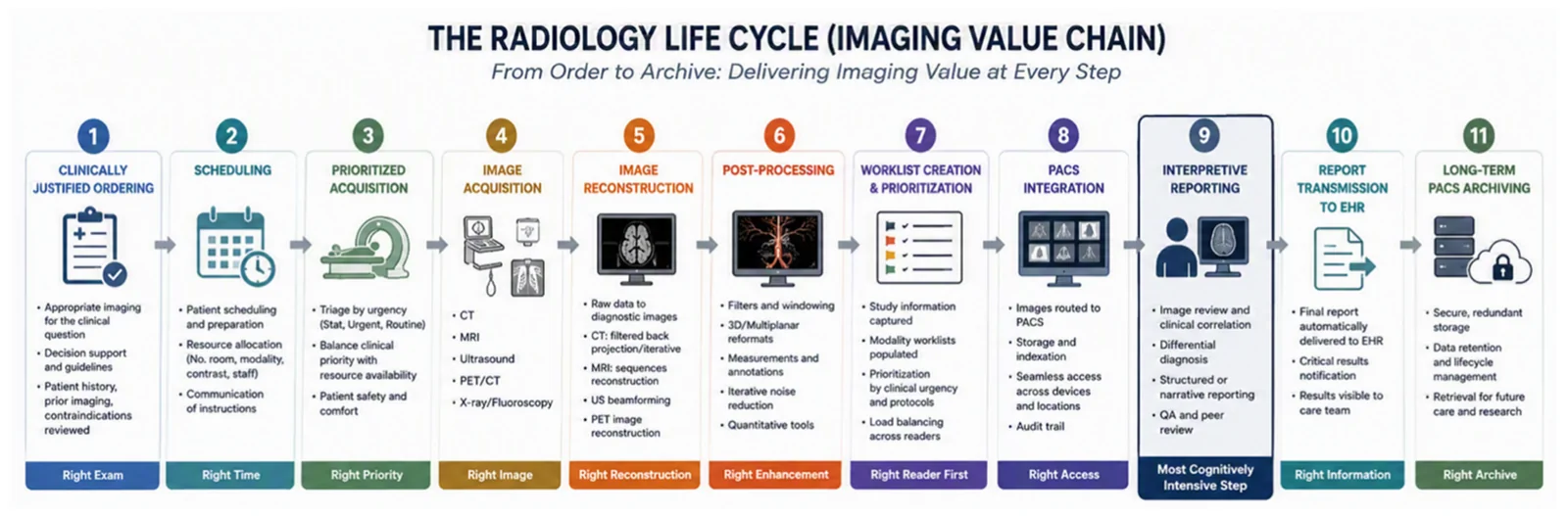
Steps of radiology life cycle.

The radiology life cycle (Figure 1)—or imaging value chain—spans every step from order to archive: clinically justified ordering, scheduling, prioritized acquisition (balancing urgency and resource availability), image reconstruction (CT, MRI, US, PET, X-ray), post-processing (filters, 3D reformats, iterative noise reduction), worklist creation and prioritization, PACS integration, interpretive reporting (the most cognitively intensive step), and final report transmission to the EHR with long-term PACS archiving. Deep learning is now inserted at nearly every node. While AI can assist with billing, scheduling, and protocoling, our focus is on image acquisition and interpretation—where AI promises enhanced diagnostic accuracy, efficiency, cost-effectiveness, and reduced liability. As we will show, however, this promise remains incompletely realized for complex oncologic tasks such as cancer-associated thromboembolism detection [4].

### Stages of Clinical-Grade AI Model Development

**Figure 2:**
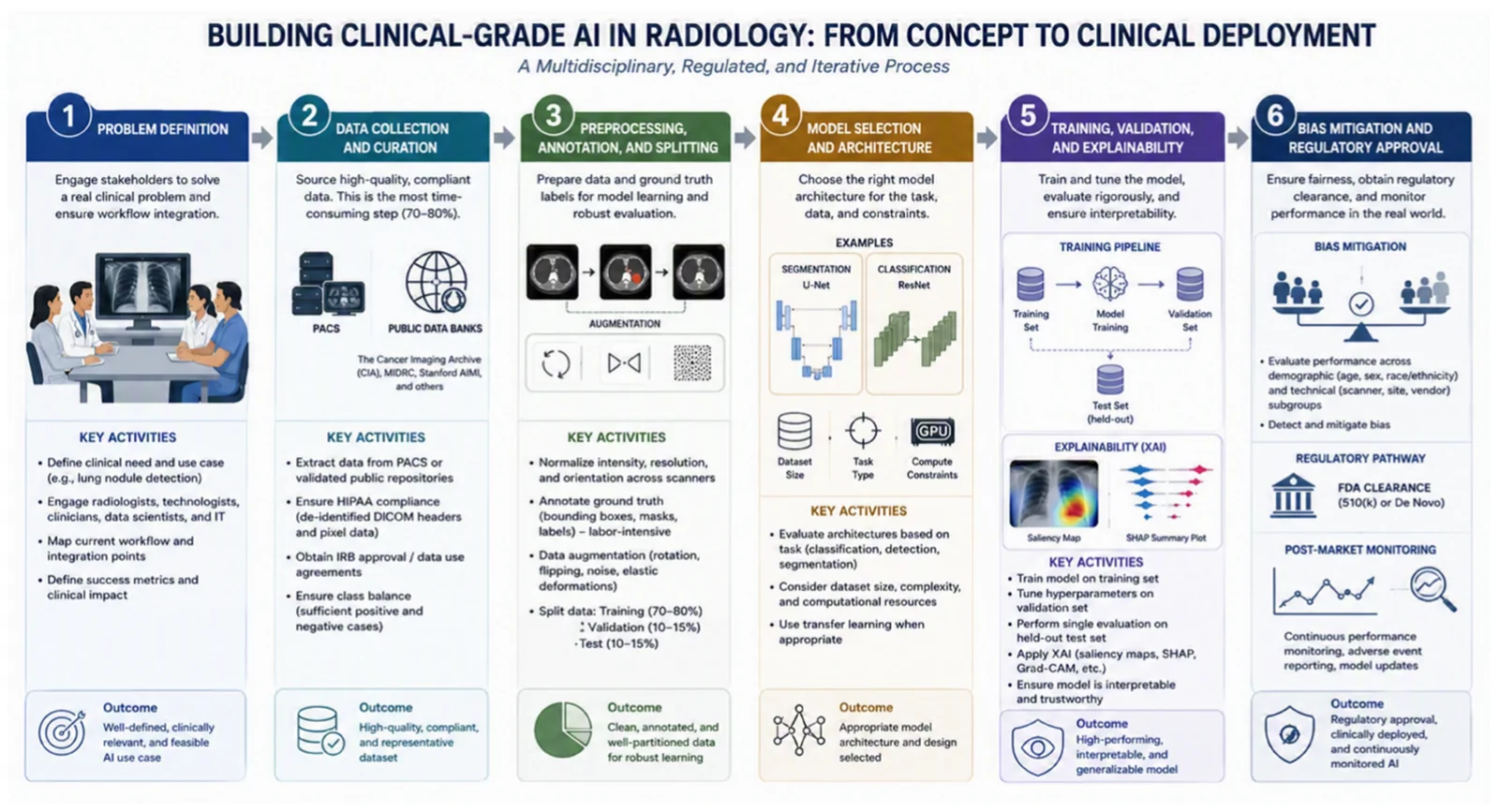
Steps for building clinical-grade AI in radiology.

Developing a clinical-grade AI model blends data science, clinical expertise, and regulatory diligence (Figure 2). It is far more than coding a neural network. The essential steps are:

1. Problem definition—Engaging radiologists, technologists, and clinicians to ensure workflow integration.
2. Data collection and curation—Sourcing from PACS or public banks (The Cancer Imaging Archive, MIDRC, Stanford AIMI). Data must be HIPAA-compliant (de-identified DICOM headers and pixel data), IRB-approved, and class-balanced (sufficient positive and negative cases). In practice, this step consumes 70–80% of project time.
3. Preprocessing, annotation, and splitting—Normalizing intensity and resolution across scanners; annotating ground truth (a rate-limiting, labor-intensive step); augmenting via rotation, flipping, or noise to improve generalization. Data are split into training (70–80%), validation (10–15%), and test (10–15%) sets.
4. Model selection and architecture—Choosing a network (e.g., U-Net for segmentation, ResNet for classification) based on task, dataset size, and computational constraints.
5. Training, validation, and explainability—Training on the training set, tuning hyperparameters on the validation set, and performing a single evaluation on the held-out test set. Explainability (XAI) methods (saliency maps, SHAP) are increasingly mandatory; black-box models are medicolegally untenable.
6. Bias mitigation and regulatory approval—Testing for bias across demographic and technical subgroups. FDA clearance (typically 510(k) or De Novo) is required before clinical deployment, followed by continuous post-market performance monitoring [5].

## 5. AI Across Radiology

### 5.1. Triaging

Radiology departments handle large volumes of imaging studies every day, with cases ranging from routine exams to potentially life-threatening cases. Triaging in radiology refers to the process of sorting and prioritizing these cases so that urgent findings are flagged and read first. Artificial intelligence (AI) enhances this process by acting as a real-time assistant, scanning images before the radiologist and assigning priority to cases with critical abnormalities such as intracranial hemorrhage on CT and pulmonary embolism on CT pulmonary angiography. This reduces time-to-diagnosis for critical conditions and minimizes delays that could affect outcomes. AI triage supports radiologists by reducing burnout, shortening turnaround times, and providing extra coverage during nights or after-hours when staffing is limited.

### 5.2. Iterative Reconstruction and Dose Reduction Tools

These tools are already inserted in radiology. They allow for reducing the dose through optimizing the scan in real-time and improving image quality (cleaning up) after its acquisition, including deep learning reconstruction, real-time dose modulation, and synthetic image generation.

### 5.3. AI and Image Interpretation

The use of artificial intelligence in **chest** imaging is most advanced in detection and diagnosis, where algorithms serve as a “second reader.” These models are trained to identify a broad range of pathologies with high accuracy, including lung nodules, interstitial lung disease, emphysema, and COPD quantification. One of the most promising applications is in diagnosing pulmonary embolism (PE) on CTPA scans [6]. AI also contributes to quantification and radiomics, where AI extracts hundreds of quantitative image features that go beyond human perception, showing potential for predicting tumor genotype, patient prognosis, and therapeutic response [7].

The most common deep learning applications in stroke detection are lesion segmentation, classification or triage, prediction of clinical outcomes, generative artificial intelligence (AI) and large language models, and reduction of imaging time or radiation dose [8]. Artificial intelligence can detect early signs of acute ischemic stroke or intracranial hemorrhage on CT and MRI scans, serving as a triage tool that flags suspected cases and can enhance communication between hospitals and comprehensive stroke centers [9,10].

AI in breast imaging assists in screening mammography, digital breast tomosynthesis (DBT), Ultrasound and MRI adjuncts, risk prediction (using imaging biomarkers + clinical data), breast density assessment, and workflow optimization [11,12,13,14]. The PRAIM study (Prospective, multicenter Observational study of an integrated AI system with live Monitoring) evaluated AI integration into real-world population-based mammography screening across 12 sites in Germany, including ~463,000 women aged 50–69. AI support increased cancer detection by 17.6% (6.7 vs. 5.7 per 1000), while recall rates remained non-inferior (37.4 vs. 38.3 per 1000). PRAIM’s significance lies in its scale, real-world setting, and hybrid workflow that balances efficiency and safety [15].

In oncologic imaging, AI algorithms are being applied to detect, characterize, and segment tumors in organs such as the liver, pancreas, kidneys, and prostate. These tools can automatically measure lesion size, volume, and growth over time, and integrate multiparametric imaging data, such as CT, MRI, and PET, to provide quantitative assessments that support treatment planning and monitoring [16,17,18]. AI models are used for automated detection of hepatic lesions, fat quantification, and fibrosis staging. It can also assist in imaging of prostate cancer [18,19,20,21]. AI offers help in triaging for incidental PE in follow-up oncologic exams within the visualized lower chest scans [22,23,24].

Artificial intelligence enhances fracture detection, providing a rapid triage system for emergency cases and helping prioritize urgent findings. [25]. Quantitative analysis is another major benefit of AI in MSK imaging, as joint space narrowing, bone mineral density, cartilage thickness, and muscle volume provide objective metrics for monitoring degenerative conditions such as osteoarthritis, osteoporosis, and sarcopenia [25,26,27].

### 5.4. Review Methodology

This is a narrative critical review. We searched PubMed and Google Scholar for English-language articles published between January 2015 and March 2026 using combinations of terms including “artificial intelligence,” “deep learning,” “machine learning,” “pulmonary embolism,” “venous thromboembolism,” “cancer,” “tumor thrombus,” “bland thrombus,” “large language model,” and “natural language processing.” We prioritized prospective studies, large retrospective cohorts, meta-analyses, and FDA regulatory documents. Reference lists of key articles were hand-searched. Given the heterogeneity of study designs and outcomes, a formal meta-analysis was not performed. Evidence was graded using a modified five-level hierarchy adapted from the Oxford Centre for Evidence-Based Medicine, specifically modified to reflect the study designs encountered in AI validation research, where large-scale prospective multicenter trials remain rare.

### 5.5. Evidence Levels [28]

- Level I: Prospective multicenter trials or meta-analyses
- Level II: Single-center prospective studies
- Level III: Large retrospective cohort studies (N > 500)
- Level IV: Case series or small retrospective studies (N < 500)
- Level V: Expert opinion, preclinical development, or algorithmic description only (Table 1).

### 5.6. Methodological Challenges and Biases in AI Studies of Cancer-Associated Thrombosis

**Figure 3:**
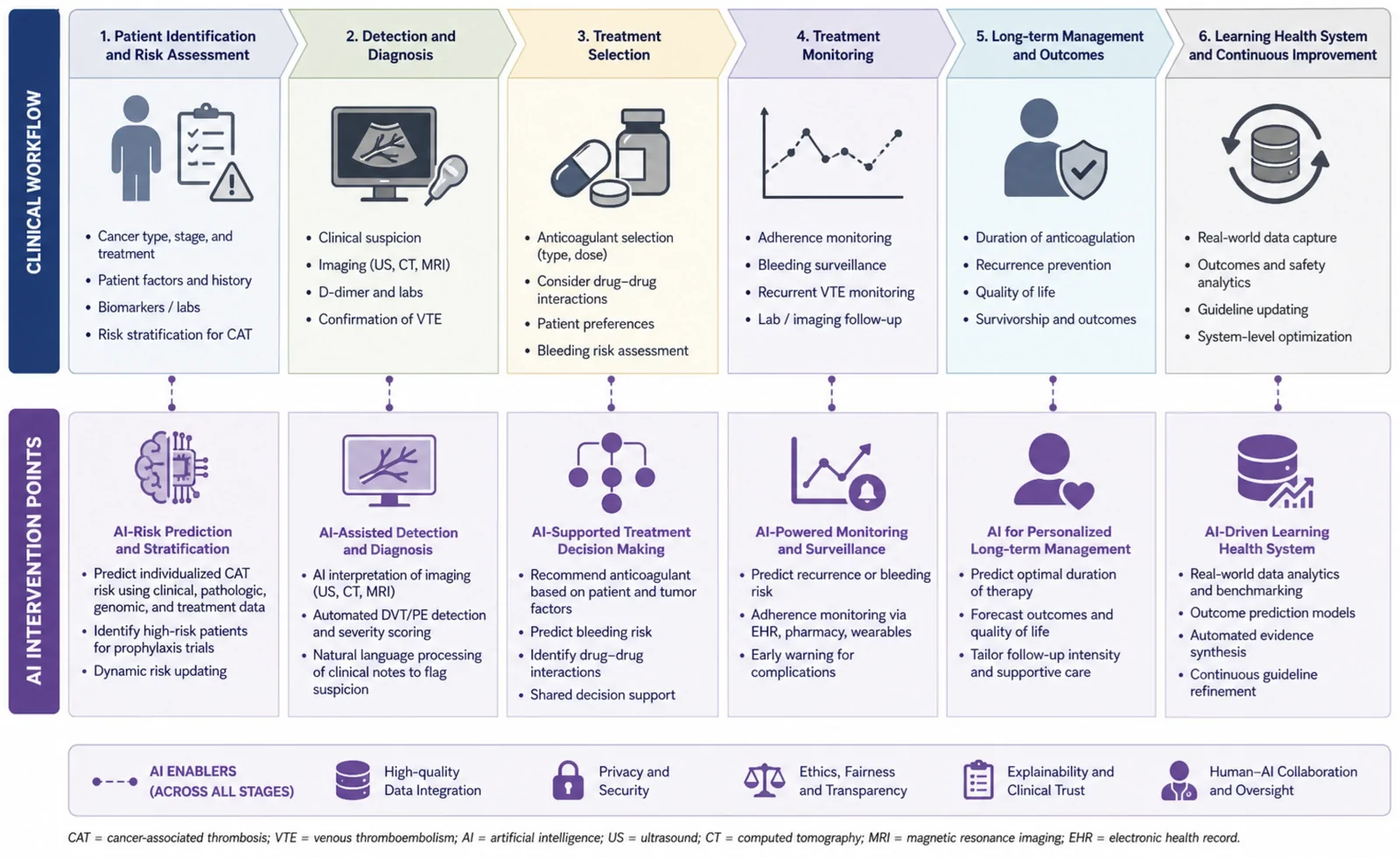
Clinical CAT workflow with AI intervention points.

Before evaluating individual clinical applications, we identify cross-cutting methodological concerns that affect AI performance in cancer populations specifically. These biases help explain the performance degradation observed when AI tools developed in general populations are applied to oncology cohorts. One of the most important is dataset shift, whereby the data encountered during real-world clinical deployment differ substantially from those used for model development (Table 2). Many AI algorithms are trained using retrospectively curated datasets acquired from a limited number of institutions under relatively standardized imaging protocols. In contrast, patients with cancer undergo imaging across diverse clinical settings with considerable variability in tumor types, disease burden, prior surgery or radiation, chemotherapy-related changes, indwelling central venous catheters, contrast timing, and imaging acquisition parameters. These differences may significantly alter the underlying data distribution, leading to deterioration in model performance when deployed in external populations. Consequently, high diagnostic accuracy reported during internal validation may not translate to comparable performance in routine clinical practice, underscoring the importance of external validation across geographically and clinically diverse oncology cohorts (Figure 3) [29].

Another fundamental challenge is class imbalance, a common characteristic of CAT datasets in which thromboembolic events represent only a small proportion of all imaging examinations. Machine learning models trained on highly imbalanced datasets may become biased toward predicting the majority class, thereby achieving deceptively high overall accuracy while demonstrating suboptimal sensitivity for detecting clinically important but infrequent events. This statistical phenomenon, rather than algorithmic failure per se, explains much of the low positive predictive value (~35%) observed in cancer-specific validation studies.

Consequently, evaluation metrics must be selected with care in low-prevalence populations. The area under the receiver operating characteristic curve should be interpreted alongside precision, recall, F1 score, calibration, and positive predictive value, particularly when assessing models intended for clinical deployment in low-prevalence populations. In addition, scanner heterogeneity remains a significant source of performance variability. Differences in CT vendor platforms, detector technology, reconstruction algorithms, and slice thickness may substantially influence image appearance. Similarly, variation in iterative reconstruction techniques, contrast administration protocols, and imaging artifacts can reduce model generalizability when algorithms are applied across institutions using different hardware or acquisition protocols [30,31,32].

Finally, institutional bias represents a persistent limitation throughout the AI development pipeline. Models derived from single-center datasets frequently learn institution-specific imaging characteristics, patient demographics, referral patterns, disease prevalence, and reporting practices that may not reflect broader clinical populations. This phenomenon can result in overly optimistic internal validation performance while masking reduced effectiveness in external healthcare systems. Selection bias, spectrum bias, and annotation bias further compound this problem, particularly when reference standards rely on retrospective radiologist interpretations that may themselves exhibit interobserver variability. Addressing these limitations will require several coordinated strategies: prospective multicenter collaborations, harmonized imaging protocols where feasible, standardized annotation methodologies, transparent reporting consistent with emerging AI-specific reporting guidelines (TRIPOD-AI, CLAIM), and routine external validation before widespread clinical implementation [32].

## 6. AI in Cancer-Associated Thromboembolism: Evidence, Gaps, and Clinical Reality

Having surveyed AI applications across radiology and identified themes relevant to thromboembolism, we now critically evaluate the evidence for AI in each phase of cancer-associated thromboembolism (CAT) management. We organize this section around three clinical tasks for which AI is being promoted: (1) incidental pulmonary embolism (PE) detection on cancer staging computed tomography (CT); (2) Differentiation between bland and tumor thrombus; (3) risk stratification for venous thromboembolism (VTE) in cancer patients; and (4) large language models. For each, we grade the available evidence using a modified hierarchy (Level I: prospective multicenter trials or meta-analyses; Level II: single prospective studies; Level III: large retrospective cohort studies; Level IV: case series or small retrospective studies; Level V: expert opinion or preclinical/algorithmic development only) [28].

### 6.1. Incidental Pulmonary Embolism Detection on Oncology Imaging

#### The Clinical Problem

Nearly all published PE detection studies exclude or underrepresent cancer patients, who have distorted vascular anatomy, variable contrast enhancement, and thrombus mimics (tumor invasion, treatment-related fibrosis) [9].

Cancer patients undergo frequent contrast-enhanced staging CT scans that visualize the pulmonary arteries. Incidental pulmonary embolism (iPE) is found in approximately 1–5% of such scans [33]. Despite this clinically significant prevalence, iPE can be overlooked by radiologists during routine surveillance imaging. Missed iPE in cancer patients carries substantial mortality risk, with studies demonstrating that untreated PE contributes to poorer prognosis in patients with malignancy [34,35].

### 6.2. AI Algorithm Characteristics

Several FDA-cleared deep learning algorithms offer automated PE detection on CT pulmonary angiography (CTPA) and, increasingly, on routine contrast-enhanced chest CT. These tools should typically run automatically in the background of picture archiving and communication systems (PACS), do not require dedicated CTPA acquisition protocols, and flag suspicious cases for radiologist review. Critically, most algorithms were not specifically trained or validated on cancer populations, despite the unique challenges this group presents.

#### Evidence Grade: Level III (Large Retrospective) for Cancer-Specific Populations

Recent large-scale retrospective studies have evaluated AI performance for iPE detection specifically in oncology patients. The most methodologically robust study to date analyzed over 3000 cancer patients undergoing routine surveillance CT at a comprehensive cancer center [34,36]. Key findings from the large cancer center study: When applied to routine oncologic staging CTs, the AI algorithm demonstrated sensitivity of 97.3% (95% CI: 85.84–99.93%), specificity of 97.74% (95% CI: 97.14–98.24%), Positive Predictive Value (PPV) 34.62%, and Negative Predictive Value (NPV) 99.97%. Of 104 AI-generated alerts, 36 patients had confirmed PE (prevalence 1.3%), while 68 alerts were false positives. Only one patient reported as negative by the AI tool was deemed to have a PE on radiologist review. False positive results were mostly due to motion, lymphadenopathy, and pulmonary pathology [34]. Other retrospective studies reported positive predictive values ranging from 10 to 83% of PE, reflecting substantial heterogeneity in the study population, PE prevalence, CT contrast timing protocol, and AI tool characteristics [35,37,38,39]. Discussing these results raises the following flags:

Approximately two-thirds of AI alerts in cancer patients are false positives, demanding careful radiologist verification. The high NPV (99.97%) provides confidence in negative results, but the low PPV risks alert fatigue.

### 6.3. Contrast Timing

A critical technical factor identified in this study was that many oncology CT scans are acquired with portal venous phase contrast injection, not in pulmonary arterial phase, posing inherent challenges for both human readers and AI algorithms. Algorithms trained on dedicated CTPA studies may not generalize to routine oncology protocols [35,40].

### 6.4. Workflow and Clinical Impact

AI tools allow for decreasing the time needed for diagnosis of iPE, which can be lowered from perhaps days in routine oncologic imaging to a few hours [34,35].

## 7. Specific Challenges in CAT Not Addressed by Current AI

### 7.1. Low Positive Predictive Value in Clinical Context

The PPV of approximately 35% means that nearly two-thirds of AI alerts are false positives. While the high NPV (>99.9%) provides confidence in negative results, the low PPV requires radiologists to review and dismiss many false alerts, potentially contributing to alert fatigue [34,36].

### 7.2. Clinically Silent Nature of AI-Detected iPEs

A 2025 study examining AI-detected iPEs across over 13,000 CT scans found that 61% of confirmed iPEs occurred in oncologic patients, but most detected emboli were segmental with no CT morphologic signs of right heart strain. Only 10% of patients with AI-detected iPEs were symptomatic. This raises a critical question: Should all AI-detected iPEs in cancer patients trigger anticoagulation, or does this risk overtreatment in clinically silent cases? The authors concluded that “integrating AI findings with morphologic and clinical criteria is crucial to avoid overtreatment” [34,41].

### 7.3. Distinction of Acute vs. Chronic PE Not Addressed

Cancer patients often have prior PE episodes. Current AI systems do not distinguish thrombus age, yet anticoagulation for chronic PE is not indicated. This limitation is particularly relevant in oncology populations where recurrent thromboembolic events are common [40,42].

### 7.4. Integration with Clinical Decision-Making

Even when AI accurately detects iPE, anticoagulation decisions require balancing thrombotic risk against bleeding risk (platelet count, brain metastases, recent surgery, drug interactions). AI provides no such clinical integration. As noted in recent radiology literature, “subtle but clinically meaningful findings can occasionally be overlooked in the context of complex oncology imaging, and AI offers a consistent mechanism to highlight abnormalities that deserve a second look” [35].

### 7.5. Clinical Bottom Line (Incidental PE Detection)

AI can identify incidental pulmonary emboli in cancer patients with high sensitivity (approximately 97%) and excellent negative predictive value (approximately >99.9%) when applied to routine oncologic staging CTs. However, the positive predictive value remains low, with most false positives attributable to motion artifacts and lymph nodes. AI implementation has demonstrated meaningful workflow benefits, reducing time-to-diagnosis from days to hours [34,35,36].

### 7.6. Key Recommendations for Clinical Practice

- AI should be used as a second reader or triage tool, not a standalone detector
- AI-negative results provide high confidence for ruling out iPE (NPV > 99.9%)
- AI-positive alerts require careful radiologist verification, with particular attention to distinguishing true emboli from mimics (lymph nodes, artifacts)
- AI findings must be integrated with clinical assessment of symptoms, thrombotic burden, and bleeding risk
- The high false-positive rate demands institutional protocols for managing AI alerts to avoid alert fatigue

## 8. AI for Tumor Thrombus vs. Bland Thrombus Differentiation

### 8.1. Clinical Problem

Differentiation of tumor thrombus (TT) from bland thrombus (BT) in major vessels is clinically critical, as the presence of TT indicates local tumor invasion, upstages disease, and contraindicates anticoagulation in favor of surgical or interventional management [43,44]. Tumor thrombus consists of viable malignant cells extending into the vascular lumen, whereas bland thrombus is composed primarily of fibrin and platelets. Conventional imaging features used to distinguish TT from BT include vessel expansion, continuity with the primary tumor, enhancement on contrast-enhanced CT or MRI, diffusion restriction, and increased FDG uptake on PET/CT [45]. However, the sensitivity and specificity of human readers for this distinction are only approximately 70–80%, with substantial interobserver variability [14]. Recent reviews emphasize that differentiating TT from BT remains particularly challenging in renal cell carcinoma, hepatocellular carcinoma, and metastatic disease, where accurate classification directly affects staging and therapeutic planning [43,46].

### 8.2. Current Evidence for AI

Radiomics offers a promising solution by extracting quantitative imaging features that are not appreciable by visual assessment alone. Although not specifically focused on venous tumor thrombus, a multicenter CT radiomics study evaluating differentiation between cardiac tumors and thrombi demonstrated that radiomic signatures can successfully distinguish neoplastic from non-neoplastic intravascular masses, suggesting potential applicability to tumor thrombus classification [47]. Similarly, MRI-based quantitative imaging approaches have shown value in differentiating neoplastic from bland portal vein thrombus in hepatocellular carcinoma through diffusion-weighted imaging (DWI) and susceptibility-weighted imaging (SWI), which can be suitable for future machine learning analysis [46,48]. “Most current evidence for AI-based thrombus differentiation relies on radiomics—hand-crafted quantitative features extracted from regions of interest—rather than deep learning with end-to-end feature learning. Deep learning approaches remain largely unexplored for this specific task, representing an additional research gap.”

The most compelling evidence for CT tumor thrombus characterization comes from a study specifically addressing portal vein thrombosis. In this retrospective study of 117 contrast-enhanced CT examinations from 109 patients with portal vein thrombosis, investigators from Massachusetts General Hospital evaluated whether CT texture analysis and thrombus attenuation measurements could differentiate neoplastic (tumor) thrombus from bland thrombus using only portal venous phase imaging [49]. Diagnostic performance was excellent, with AUCs of 0.97 for mean value of positive pixels, 0.93 for entropy, and 0.99 for a combined model incorporating both parameters, outperforming thrombus attenuation alone (AUC 0.91) and substantially exceeding subjective radiologist assessment (AUC 0.61). These findings demonstrate that quantitative CT texture analysis and attenuation measurements can reliably distinguish neoplastic from bland thrombi on a single portal venous phase CT examination, highlighting the potential of quantitative imaging biomarkers and AI-related radiomic techniques for thrombus characterization [49].

## 9. Future Directions for AI Model Development

Future AI models may benefit from integrating contrast-enhancement characteristics, texture-based radiomic features, diffusion-weighted MRI metrics, and PET metabolic activity parameters within multimodal architectures. However, progress is constrained by small datasets, lack of multicenter validation, and the rarity of pathologically confirmed cases [46].

### 9.1. Limitations and Gaps

This domain remains far from clinical readiness. Notable gaps in the literature include:

1. Absence of large-scale deep learning studies specifically targeting TT versus BT classification [14]. Most AI research involving thrombus characterization has focused on segmentation, tumor detection, or differentiation between benign and malignant masses rather than vascular tumor extension.
2. Lack of standardized imaging protocols across institutions limits generalizability of radiomic features [14,17].
3. Limited ground truth—histopathological confirmation of thrombus type is infrequently obtained, as biopsy of intravascular material carries procedural risk. Most studies rely on imaging follow-up or clinical response to anticoagulation as reference standards, introducing verification bias [14].
4. No FDA-cleared tools—all work remains investigational. No commercial AI product offers thrombus characterization as a clinical output.

### 9.2. Clinical Bottom Line

Evidence Grade: IV (small retrospective series, single institution). AI and radiomics demonstrate promise for distinguishing tumor thrombus from bland thrombus in retrospective studies, with excellent diagnostic performance (AUC 0.97–0.99) in proof-of-concept analyses [17]. However, all evidence is retrospective and single-center. No FDA-cleared tool exists, and prospective validation is absent. This represents a potentially high-impact area for future investigation, particularly given the increasing availability of contrast-enhanced CT datasets from oncologic patients and the importance of accurate thrombus characterization for staging and treatment planning [14]. Radiologists should not rely on AI for thrombus characterization.

### 9.3. Risk Prediction Models for CAT

#### 9.3.1. Comparison with Khorana Score

The Khorana score remains the guideline-recommended tool for identifying cancer patients at high risk of venous thromboembolism (VTE) [50]. A comprehensive narrative review and network meta-analysis of 28 studies, published in December 2025, evaluated 12 modified versions of the Khorana score. However, none of the modified Khorana scores was significantly superior to the original Khorana score in pairwise meta-analysis. Furthermore, 96.4% of included studies were identified as having a high risk of bias, and calibration performance was rarely reported. The authors concluded that “the Khorana score and its modifications did not currently meet clinically desirable standards of evidence quality and predictive accuracy” [50]. Machine learning models trained on electronic health record data have demonstrated improved discrimination for CAT risk compared with the Khorana score, a static baseline-only risk model. A narrative review found that ML approaches, particularly gradient boosting algorithms, generally outperform the Khorana score across datasets, though the magnitude of improvement varies substantially by cohort [51].

#### 9.3.2. Multimodal and Imaging-Based Approaches

Beyond clinical data alone, multimodal approaches combining imaging features with clinical parameters have shown promise. A deep learning model incorporating CT pulmonary angiography features, clinical data, and Pulmonary Embolism Severity Index (PESI) scores demonstrated improved survival prediction for PE patients compared to PESI scores alone. However, this study was not conducted in a cancer-specific population [52].

For cancer patients specifically, radiomics-based models extracting quantitative features from tumor morphology on baseline staging CTs have been evaluated. In pancreatic cancer patients (N = 312), a CT radiomics model achieved an AUC of 0.74 for VTE prediction, compared with 0.61 for the Khorana score. In lung cancer patients (N = 458), a PET/CT radiomics model achieved an AUC of 0.79 with a net reclassification improvement of 32%. A multicenter study in colorectal cancer (N = 523, with external validation N = 212) reported that a deep learning model achieved an AUC of 0.76, and combining the Khorana score with radiomics improved performance to an AUC of 0.81 [53,54,55].

#### 9.3.3. Prospective Observational Data

The PARADIGM study, a prospective observational study of 1204 patients with mixed solid tumors, evaluated a combined CT radiomics and clinical model for VTE prediction. The model achieved an AUC of 0.77 and was well-calibrated in the validation cohort, representing the highest level of evidence currently available for AI-based CAT risk stratification [56].

#### 9.3.4. Additional Limitations

1. High risk of bias: Most models developed and tested on data from one healthcare system; external validation across diverse populations is rare [53,55].
2. Cancer type heterogeneity: Features predicting VTE in pancreatic cancer (desmoplastic, hypovascular) likely differ from renal cell carcinoma (hypervascular) or lymphoma. Pooled models may not generalize [53,56].
3. Temporal instability: Tumor size, necrosis, and vasculature change with therapy. Baseline models may not reflect risk after chemotherapy initiation [54].
4. Cost-effectiveness unproven: Even if AI improves prediction, the added cost of implementation versus clinical scores alone has not been evaluated [51].

Despite these promising metrics, no study has demonstrated that AI-guided prophylaxis reduces VTE or improves survival. The fundamental question remains: “Does telling the oncologist about AI-predicted risk change management and improve outcomes?” This question has not been tested in any prospective interventional trial [51,56].

#### 9.3.5. Clinical Bottom Line (Risk Prediction)

Evidence Grade: II–III (prospective observational to large retrospective). ML models modestly outperform the Khorana score (AUROC improvement approximately 0.10–0.15). However, prospective validation is limited to a single study, and no outcome trials have been conducted. These methods remain investigational and should not guide clinical decision-making [51,56].

## 10. AI Reporting and Validation Standards

The rapid expansion of artificial intelligence (AI) in medical imaging has highlighted the need for standardized reporting and rigorous validation to ensure transparency, reproducibility, and clinical applicability. Historically, many AI studies have been limited by incomplete reporting of dataset characteristics, inadequate external validation, inconsistent performance metrics, and insufficient descriptions of model development, making it difficult to assess methodological quality or reproduce published results. To address these challenges, several AI-specific reporting guidelines have been developed:

**TRIPOD-AI:** The Transparent Reporting of a Multivariable Prediction Model for Individual Prognosis or Diagnosis–Artificial Intelligence (TRIPOD-AI) statement extends the original TRIPOD guideline by providing recommendations for transparent reporting of AI-based diagnostic and prognostic prediction models, including detailed descriptions of data preprocessing, model development, validation strategies, handling of missing data, and performance assessment. For prospective interventional studies evaluating AI in clinical practice.

**CONSORT-AI:** The Consolidated Standards of Reporting Trials–Artificial Intelligence (CONSORT-AI) extension supplements the CONSORT guideline by emphasizing reporting of the AI intervention, human–AI interaction, algorithm versioning, integration into clinical workflow, and protocol deviations unique to AI-assisted randomized controlled trials.

**CLAIM:** Within medical imaging, the Checklist for Artificial Intelligence in Medical Imaging (CLAIM) provides a radiology-specific framework encompassing all stages of AI development, from dataset acquisition and annotation to model architecture, reference standards, statistical analysis, external validation, and assessment of clinical utility. Together, these reporting standards promote methodological rigor, facilitate independent validation, improve reproducibility, and enable more meaningful comparisons across studies. As AI applications in cancer-associated thromboembolism continue to evolve, adherence to TRIPOD-AI, CONSORT-AI, and CLAIM should become an essential expectation for future investigations, ensuring that proposed algorithms are evaluated not only for technical performance but also for robustness, transparency, generalizability, and clinical relevance before widespread implementation [57,58,59,60,61].

### 10.1. Implementation Barriers: Economic, Technical, and Workflow Considerations

Despite growing interest in artificial intelligence (AI) for cancer-associated thromboembolism (CAT), widespread clinical implementation will depend not only on diagnostic performance but also on economic feasibility and successful integration into existing healthcare infrastructure. The acquisition and maintenance of AI systems require substantial financial investment, including software licensing, hardware upgrades, cloud computing resources, cybersecurity measures, information technology support, and ongoing software updates. These costs may be particularly burdensome for smaller hospitals, community oncology practices, and resource-limited healthcare systems, potentially widening disparities in access to advanced AI technologies. Beyond direct costs, technical integration presents additional challenges. Implementation often necessitates integration with picture archiving and communication systems (PACS), radiology information systems (RIS), electronic health records (EHR), and clinical notification platforms. Achieving seamless interoperability across these systems remains technically challenging because of variability in vendor platforms, proprietary software architectures, and institutional workflows [62].

The economic value of AI in CAT also remains uncertain because few studies have evaluated cost-effectiveness using clinically meaningful endpoints. Although AI-assisted incidental pulmonary embolism (iPE) detection has consistently demonstrated reductions in time-to-notification and may improve radiologist workflow efficiency, these operational benefits do not necessarily translate into lower healthcare expenditures or improved patient outcomes. Cost-effectiveness analyses should account not only for implementation costs but also for downstream consequences of AI performance, including unnecessary diagnostic workup and anticoagulation resulting from false-positive findings, delayed diagnosis associated with false-negative results, additional radiologist review time, and potential reductions in medicolegal risk or hospital length of stay. For example, a false-positive AI alert for iPE in a thrombocytopenic cancer patient may trigger unnecessary anticoagulation, leading to bleeding complications, extended hospitalization, and additional imaging costs that must be weighed against the benefits of earlier PE detection in true-positive cases. Importantly, demonstrating improved diagnostic accuracy alone is insufficient to justify widespread adoption; AI must ultimately demonstrate value by improving patient-centered outcomes while remaining economically sustainable [63].

Technical implementation presents additional challenges beyond financial considerations. AI models require continuous performance monitoring to detect degradation resulting from dataset shift, evolving imaging protocols, software updates, and changes in patient populations. Consequently, healthcare institutions must establish governance frameworks for model validation, quality assurance, version control, cybersecurity, and periodic recalibration before and after deployment. Dedicated personnel—including radiologists, information technology specialists, clinical informaticians, data scientists, and hospital administrators—are often required to oversee these processes, creating additional operational costs that are rarely considered in initial implementation analyses. Moreover, AI-generated outputs must be presented in a manner that integrates naturally into radiologists’ workflows without contributing to alert fatigue or increasing interpretation time. Poor user interface design or excessive false-positive notifications may diminish clinician confidence, reduce adoption, and ultimately negate potential workflow benefits [64,65,66,67].

Future studies should therefore move beyond reporting diagnostic accuracy alone and incorporate comprehensive health economic evaluations alongside prospective implementation research. Such investigations should assess cost-effectiveness, workflow efficiency, resource utilization, reimbursement considerations, clinician acceptance, and patient-centered outcomes across diverse healthcare settings. Demonstrating that AI provides measurable clinical value while remaining economically viable and technically scalable will be essential before its widespread adoption in diagnosis, risk stratification, and management. These considerations are reflected in the research roadmap outlined below, which prioritizes health economic evaluations alongside prospective outcome trials [68,69].

### 10.2. Medicolegal Liability of AI-Assisted Radiology

The increasing integration of artificial intelligence (AI) into clinical practice raises important medicolegal questions regarding liability when AI-assisted recommendations contribute to diagnostic errors in cancer-associated thromboembolism (CAT). This section examines the allocation of liability among physicians, institutions, and developers; identifies CAT-specific medicolegal risks; and proposes institutional safeguards for clinical deployment. Although AI systems are often marketed as clinical decision-support tools rather than autonomous diagnostic systems, false-positive or false-negative outputs may influence physician decision-making and ultimately affect patient outcomes. In the context of incidental pulmonary embolism (iPE), for example, a false-positive AI alert may prompt unnecessary anticoagulation, exposing patients to avoidable bleeding complications, additional diagnostic testing, increased healthcare costs, and psychological distress. Conversely, overreliance on AI may contribute to diagnostic anchoring, whereby clinicians are disproportionately influenced by algorithm-generated findings despite conflicting clinical or imaging evidence. These scenarios highlight that the medicolegal implications of AI extend beyond algorithm performance alone and encompass the interaction between clinicians, healthcare institutions, and AI developers [70].

At present, no universally accepted legal framework assigns liability for AI-related diagnostic errors in cancer-associated thromboembolism. In most jurisdictions, responsibility for clinical decision-making continues to rest primarily with the treating physician or interpreting radiologist, who is expected to independently evaluate AI-generated recommendations within the broader clinical context. Consequently, AI should be regarded as an adjunctive tool that informs—but does not replace—professional judgment. Healthcare institutions also bear responsibility for appropriate implementation, including validation of AI performance within local practice environments, clinician education, quality assurance, cybersecurity, and ongoing post-deployment monitoring. Meanwhile, AI developers may be subject to product liability if harm results from defects in software design, inadequate validation, failure to disclose known limitations, or insufficient post-market surveillance, although the legal standards governing such claims continue to evolve. Given these uncertainties, institutions adopting AI for CAT should establish clear governance frameworks defining clinician oversight, documentation of AI use, mechanisms for reporting algorithm-related adverse events, and periodic auditing of real-world performance. Until comprehensive regulatory and legal standards mature, maintaining meaningful human oversight remains not only a clinical necessity but also an essential component of medicolegal risk mitigation [71].

We recommend that institutions deploying AI for CAT develop explicit protocols that: (1) define the radiologist’s responsibility to independently verify all AI-positive findings; (2) require documentation of AI use in the radiology report; (3) establish communication pathways between radiologists and oncologists when AI-detected PE is confirmed; and (4) mandate integration of bleeding risk assessment before anticoagulation decisions. From a regulatory perspective, FDA guidance on clinical decision-support software, updated in January 2026, explicitly clarifies that software functions analyzing a radiologist’s clinical findings to generate proposed summaries—including diagnostic recommendations based on clinical guidelines—may be excluded from medical device regulation, provided that a healthcare professional reviews, revises, and finalizes the output. This framework reinforces the primacy of human oversight while creating a potential regulatory pathway for AI-assisted decision support in CAT, though CAT-specific validation in cancer populations remains an essential prerequisite that has not yet been met [60,72,73,74].

## 11. Large Language Models: Emerging Evidence and Persistent Gaps

### 11.1. Proposed Applications and Recent Advances

Large language models (LLMs) have been proposed for multiple applications in radiology, including clinical history integration, structured report generation, knowledge retrieval, and patient communication. [75,76].

A recent systematic review examining LLM-generated radiology reports (nine studies published between January 2015 and February 2025) found that fine-tuned models demonstrated better alignment with expert evaluations and achieved higher performance on natural language processing metrics compared to base models. However, the review also identified persistent challenges: all evaluated LLMs showed hallucinations, misdiagnoses, and inconsistencies, leading to the conclusion that “limitations in diagnostic accuracy and hallucinations necessitate human oversight” [77].

While no study has specifically evaluated LLMs for CAT detection or risk prediction, recent research has applied natural language processing (NLP)—a related but distinct technology—to CAT management. In a large multicenter study of 21,227 cancer patients with VTE receiving anticoagulants across nine Spanish hospitals, researchers used NLP and machine learning to develop a predictive model for major bleeding within six months of VTE diagnosis. This study is significant for two reasons: (1) it represents the first large-scale application of NLP to a CAT-specific outcome (major bleeding in anticoagulated cancer patients), and (2) it demonstrates that text-based AI approaches can extract clinically meaningful information from unstructured EHR data in this population [27,28]. However, the authors acknowledge that the predictive performance remains “moderate,” and the study did not evaluate generative LLMs (e.g., GPT-4) specifically [78].

The hallucination risk identified in general radiology LLM studies [26] is further substantiated by research in other high-stakes clinical domains. A rigorous evaluation of three generative LLMs (GPT-4o, Claude 3 Sonnet, Gemini Ultra 1.0) for stroke care—a domain with similar clinical urgency to CAT—found that all models performed below clinical competency thresholds across prevention, diagnosis, treatment, and rehabilitation stages. Key findings included that the treatment stage exhibited the weakest performance (48.2–57.8) across all stages and no model prompt passed successfully through all domains [79].

### 11.2. Recommendations

Please refer to Table 3, Table 4 and Table 5 for recommendations for radiologists and oncologists, AI developers, and regulatory bodies.

## 12. Summary of Principal Findings

This review had two aims: (1) to provide a practical overview of FDA-approved AI tools in radiology, and (2) to critically evaluate AI evidence for cancer-associated thromboembolism. For general radiology, we found that while over 1000 FDA-cleared AI tools exist, widespread clinical adoption remains limited due to integration challenges, lack of radiologist familiarity, and sparse prospective validation. For CAT specifically, four key findings emerged:

## 13. Key Points

- Incidental PE detection: AI demonstrates high sensitivity (~97%) and NPV (>99.9%) but low PPV (~35%); use only as a second reader or triage tool with mandatory radiologist verification of all positive alerts [33,34,35].
- Tumor vs. bland thrombus differentiation: Radiomics-based proof-of-concept studies show promise (AUC 0.97–0.99) but remain investigational; no FDA-cleared tools exist, and all evidence is retrospective and single-center [46,47].
- VTE risk prediction: Machine learning models modestly outperform the Khorana score (AUC improvement 0.10–0.15), but no prospective interventional trial has demonstrated that AI-guided prophylaxis reduces VTE or improves survival [53,54].
- Large language models: Generative LLMs have no CAT-specific validation, and evidence from stroke care demonstrates subclinical performance with persistent hallucination risks unacceptable for anticoagulation decisions [75,77].
- Cross-cutting finding: AI performance consistently degrades when moving from general populations to cancer-specific cohorts; cancer-specific validation and prospective outcome trials are needed before clinical deployment expands beyond assistive PE detection [78,79].

## 14. Conclusions

The benefits of AI in radiology are undeniable. However, critical scrutiny and mandatory human oversight are mandatory when handling AI tools and suggestions. Returning to our central question: Can artificial intelligence really help, especially in cancer-associated thromboembolism? The answer depends on the task—and on the standards of evidence one accepts.

For incidental PE detection on oncology staging CT, AI can help—but only as a second reader or triage tool, not as a standalone detector. The low positive predictive value (∼35%) means nearly two-thirds of alerts are false positives, demanding careful radiologist verification. AI reduces time-to-diagnosis from days to hours, representing a meaningful workflow benefit. Yet no study has shown that AI-detected iPE followed by anticoagulation improves survival or reduces recurrent VTE.

For differentiation of tumor thrombus from bland thrombus, AI cannot help in clinical practice today. Proof-of-concept radiomics studies show promise, but all evidence is retrospective and single-center. No FDA-cleared tool exists.

For VTE risk stratification, AI may help in the future, but not yet. Machine learning models modestly outperform the Khorana score, but the Khorana score itself demonstrates poor discriminative ability. No prospective interventional trial has shown that AI-guided prophylaxis reduces VTE or improves survival.

For large language models, AI cannot help at all. No study has evaluated LLMs for any CAT-specific task. Evidence from stroke care demonstrates subclinical performance and persistent hallucination risks that are unacceptable for anticoagulation decisions.

Across all four domains, a consistent pattern emerges: AI’s performance degrades significantly when moving from general populations to cancer-specific cohorts. The heterogeneity of cancer patients—variable anatomy, contrast protocols, prior treatments, and comorbidities—breaks the assumptions on which most AI models were trained.

## Figures and Tables

**Table 1:** Summary Evidence Grades for AI in CAT.

**Application**	**Evidence Level**	**Data**	**Key Limitation**	**Clinical Readiness**
Incidental PE detection on oncology CT	III (large retrospective)	Sensitivity 85–97%, Specificity 95–99%, PPV 10–83%, NPV > 99%.	PPV ~35%; false positives from artifacts/lymph nodes	Conditional: second reader only
Tumor thrombus vs. bland thrombus differentiation	IV (small retrospective)	AUC 0.93–0.99 (single-center, radiomics-based only).	No FDA-cleared tools; pathologic correlation often missing	Investigational only
VTE risk prediction	II–III (prospective observational; no RCTs)	AUC improvement over Khorana score 0.10–0.15; best-performing combined model AUC 0.81	Modest improvement over Khorana score; no outcome trials	Research only
Large language models for CAT	V (no CAT-specific data)	No quantitative CAT-specific performance data available.	Hallucination risk; no regulatory approval	Not ready
**Application**	**Evidence Level**		**Key Limitation**	**Clinical Readiness**
Incidental PE detection on oncology CT	III (large retrospective)		PPV ~35%; false positives from artifacts/lymph nodes	Conditional: second reader only
Tumor thrombus vs. bland thrombus differentiation	IV (small retrospective)		No FDA-cleared tools; pathologic correlation often missing	Investigational only
VTE risk prediction	II–III (prospective observational; no RCTs)		Modest improvement over Khorana score; no outcome trials	Research only
Large language models for CAT	V (no CAT-specific data)		Hallucination risk; no regulatory approval	Not ready

CAT: cancer-associated thromboembolism, CT: computerized tomography, FDA: Food and Drug Administration, PE: pulmonary embolism, PPV: positive predictive value & VTE: venous thromboembolism. N.B. formal pooling was not performed due to study heterogeneity, and ranges are presented to illustrate the evidence gap.

**Table 2:** Common sources of bias and methodological challenges in AI studies of cancer-associated thrombosis.

Bias/Challenge	Description	Example in CAT AI	Potential Impact	Mitigation Strategies
Dataset shift (distribution shift)	Training and deployment populations differ	Model trained at tertiary cancer centers deployed in community hospitals	Reduced generalizability and diagnostic performance	Multi-center training, external validation, prospective monitoring
Low-prevalence class imbalance	Positive cases are much less common than negative cases	Incidental PE present in only a small fraction of oncology CT examinations	High overall accuracy but reduced sensitivity and PPV	Oversampling, focal loss, balanced sampling, cost-sensitive learning
Scanner heterogeneity	Imaging varies across vendors and acquisition protocols	Different CT vendors, slice thicknesses, reconstruction kernels, or contrast protocols	Learned imaging features fail to generalize	Image harmonization, domain adaptation, data augmentation
Institutional bias	Models learn site-specific rather than disease-specific patterns	Scanner identifiers or institutional workflow characteristics correlate with thrombosis	Excellent internal validation but poor external performance	Multi-institutional datasets and independent external validation
Selection bias	Training cohort is not representative of the target population	Academic centers overrepresent advanced malignancies	Reduced applicability to community practice	Representative recruitment across diverse practice settings
Spectrum bias	Disease severity differs between training and clinical populations	Training contains predominantly large PE while practice includes subtle incidental PE	Reduced detection of mild disease	Include the full spectrum of disease severity during training
Annotation bias	Reference labels are inconsistent or imperfect	Interobserver disagreement for isolated subsegmental PE	Noisy ground truth reduces model accuracy	Consensus annotation, multiple expert readers, adjudication
Verification bias	Only selected patients undergo confirmatory testing	Symptomatic patients preferentially receive reference-standard imaging	Overestimation of diagnostic accuracy	Standardized verification protocols and complete follow-up
Temporal bias	Clinical practice and imaging evolve over time	Older CT acquisition protocols differ from current practice	Progressive performance degradation after deployment	Periodic retraining and temporal validation
Confounding bias	Model learns correlated but clinically irrelevant features	Chemotherapy ports become a surrogate marker for thrombosis	Spurious associations and poor robustness	Explainability analyses, causal modeling, feature auditing
Demographic bias	Unequal representation across patient subgroups	Underrepresentation of younger patients or racial/ethnic minorities	Unequal diagnostic performance across populations	Diverse datasets and subgroup-specific performance evaluation
Cancer-type bias	Certain malignancies dominate the training cohort	Lung cancer overrepresented relative to pancreatic or hematologic malignancies	Limited generalizability across cancer types	Stratified sampling and cancer-specific validation
Treatment bias	Treatment-related imaging findings influence predictions	Chemotherapy- or radiation-induced imaging changes drive model decisions	Reduced transportability across treatment settings	Incorporate treatment metadata and external validation
Label leakage	Information unavailable at inference is inadvertently included during training	Radiology report text contributes to model inputs	Artificially inflated performance estimates	Strict separation of input features and outcome labels
Prevalence bias	Disease prevalence differs across institutions	Referral cancer centers have substantially higher CAT prevalence	Poor probability calibration in new populations	Local recalibration and prospective validation

**Table 3:** Clinical Recommendations for Radiologists and Oncologists.

Subject	Recommendation	Evidence Grade	Rationale
AI for incidental PE	Second reader only or triage	III	High sensitivity (97%) but low PPV (35%); NPV > 99.9% supports ruling out PE
AI for PE in routine oncology exam.	Verify all positive findings against motion artifact or lymph nodes	III	68 of 104 alerts were false positives; 32% from motion, 16% from lymph nodes
AI use for initiation of anticoagulation in iPE	Don’t	III	Clinically silent iPEs (90% of AI-detected cases) may not require anticoagulation
Risk prediction models and clinical decision	Not ready	II–III	No prospective interventional trial has demonstrated improved outcomes
LLM for cancer related clinical task	not recommended	V	No CAT-specific validation; hallucination risks unacceptable for anticoagulation decisions.

AI: artificial intelligence, CAT: cancer-associated thromboembolism, LLM: large language model, iPE: incidental pulmonary embolism, NPV: negative predictive value, PE: pulmonary embolism, PPV: positive predictive value.

**Table 4:** Recommendations for AI Developers.

Recommendation	Evidence Grade	Rationale
Train and validate algorithms on cancer patient cohorts	III	Current algorithms trained on ED/general populations; PPV drops to 35% in cancer
Report cancer-specific subgroup performance in PE detection studies	III	Less than 5% of patients in most studies have cancer; subgroup analysis rarely reported
Develop algorithms that distinguish acute from chronic PE and bland from tumor thrombus	IV	Current AI cannot make these distinctions, yet management differs radically
Integrate clinical variables into PE detection outputs	V	Anticoagulation decisions require balancing thrombotic and bleeding risk

AI: artificial intelligence, ED: emergency department, PE: pulmonary embolism, PPV: positive predictive value.

**Table 5:** Recommendations for Policymakers and Regulatory Bodies.

Recommendation	Evidence Grade	Rationale
Require cancer-specific validation for FDA clearance of AI tools intended for CAT	III	Current 510(k) pathway does not mandate subgroup validation
Mandate prospective outcome trials (not just diagnostic accuracy) for CAT AI tools	III	No study has shown AI improves CAT mortality, recurrence, or bleeding outcomes
Develop standards for reporting AI performance in cancer populations (e.g., STARD-AI extension)	V	Current reporting lacks calibration, cancer subgroup analyses, and false positive characterization

AI: artificial intelligence, CAT: cancer-associated thromboembolism, FDA: US Food and Drug Administration.

## Data Availability

No new data were created or analyzed in this study. Data sharing is not applicable to this article.

## Abbreviations

AI	Artificial Intelligence
AUC	Area Under the Curve
BI-RADS	Breast Imaging Reporting and Data System
BT	Bland Thrombus
CAD	Computer-Aided Diagnosis
CAT	Cancer-Associated Thromboembolism
CI	Confidence Interval
CNNs	Convolutional Neural Networks
COPD	Chronic Obstructive Pulmonary Disease
CT	Computed Tomography
CTPA	Computed Tomography Pulmonary Angiography
DBT	Digital Breast Tomosynthesis
DL	Deep Learning
DWI	Diffusion-Weighted Imaging
EHR	Electronic Health Record
EMR	Electronic Medical Record
FDA	U.S. Food and Drug Administration
FDG	Fluorodeoxyglucose
GPUs	Graphics Processing Units
HIPAA	Health Insurance Portability and Accountability Act
iPE	Incidental Pulmonary Embolism
IRB	Institutional Review Board
LI-RADS	Liver Imaging Reporting and Data System
LLMs	Large Language Models
LVOs	Large Vessel Occlusions
MIDRC	Medical Imaging and Data Resource Center
ML	Machine Learning
MRI	Magnetic Resonance Imaging
MSK	Musculoskeletal
NLP	Natural Language Processing
NPV	Negative Predictive Value
PACS	Picture Archiving and Communication System
PE	Pulmonary Embolism
PESI	Pulmonary Embolism Severity Index
PET	Positron Emission Tomography
PI-RADS	Prostate Imaging Reporting and Data System
PPV	Positive Predictive Value
RCTs	Randomized Controlled Trials
RIS	Radiology Information System
RSNA	Radiological Society of North America
SHAP	Shapley Additive exPlanations
SWI	Susceptibility-Weighted Imaging
TT	Tumor Thrombus
US	Ultrasound
VTE	Venous Thromboembolism
XAI	Explainable Artificial Intelligence
